# Translational profiling identifies sex‐specific metabolic and epigenetic reprogramming of microglia in cerebral amyloidosis models with an antibiotic‐altered gut microbiome

**DOI:** 10.1002/alz.084789

**Published:** 2025-01-03

**Authors:** Shabana M Shaik

**Affiliations:** ^1^ The University of Arizona ‐ Tucson, Tucson, AZ USA

## Abstract

**Background:**

Host commensal gut microbes are shown to be crucial for microglial maturation, and functions that involve innate immune responses to maintain brain homeostasis. Sex has a crucial role in the incidence of neurological diseases with females showing higher progression of AD compared with males. Transcriptomics has been a powerful tool for the characterization of microglial phenotypes however, there is a large gap in relating to their functional protein abundances. Here, we generated ‘APPPS1‐21‐CD11br’ a translating ribosome affinity purification (TRAP) mice model and assessed the influence of gut microbiome on microglial protein networks during their phenotypic transition in a sex‐specific manner.

**Methods:**

APPPS1‐21 mice were crossed with WT‐CD11br mice wherein a CD11b‐promoter drives expression of ribosomal protein L10a (RpL10) that is fused to FLAG/EGFP at the amino‐terminus to generate APPPS1‐21‐CD11br transgenic mice. Six groups of mice that included WT‐CD11br, antibiotic (ABX) or vehicle‐treated APPPS1‐21‐CD11br male and female were sacrificed at 7‐weeks of age (n = 15/group) and used for immunoprecipitation of microglial polysomes from cortical homogenates using anti‐FLAG antibody. Liquid chromatography coupled to tandem‐mass‐spectrometry (LC‐MSMS) and label‐free quantification was used to identify newly synthesized peptides extracted from polysomes.

**Results:**

Proteomic analysis reveal that Aβ‐induced microglial activation resulted in increased FcgR‐mediated‐phagocytosis and actin organization in male and female AD mice respectively. ABX‐treatment resulted in substantial remodeling of epigenetic landscape, leading to a metabolic shift that accommodates the increased bioenergetic and biosynthetic demands associated with microglial polarization in a sex‐specific manner. While microglia in ABX‐treated male mice exhibited a metabolic shift towards ketogenesis to promote neuroprotective phenotype mediating lysosomal Aβ clearance, microglia in ABX‐treated female mice exhibited persistent mitochondrial dysfunction and impaired clearance associated with inflammatory phenotypes. Targeted metabolomics show sex‐specific changes in immunomodulatory gut metabolites mediated by ABX‐treatment.

**Conclusions:**

Our studies provide first snapshot of dynamic translational state of microglia in cerebral Aβ‐amyloidosis models with an altered gut‐microbiome. Gut metabolites can support microglial metabolic plasticity to modulate immune responses and amyloid clearance in a sex‐specific manner. Reprogramming of microglial metabolism could be a promising strategy for restoring their physiological functions in a manner that might halt the progression of AD.

